# Familial Cognitive Dissonance in the Context of Brain Death: A Case Report and Theoretical Analysis

**DOI:** 10.7759/cureus.100039

**Published:** 2025-12-24

**Authors:** Maria Kitsiou, Polyxeni Tsiokanou

**Affiliations:** 1 Department of Nursing, University of Ioannina, Ioannina, GRC

**Keywords:** brain death, cognitive dissonance, denial, family response, organ donation

## Abstract

Brain death, defined as the irreversible cessation of all brain and brainstem functions, is legally and medically recognized as death, yet families often struggle to accept it. We report the case of a 65-year-old man admitted to the intensive care unit with a massive intracerebral hemorrhage. Despite maximal interventions, he progressed to brain death confirmed by neurological examination and apnea testing. While the patient’s wife and daughter gradually accepted the diagnosis, his son reacted with disbelief, anger, and persistent denial, rejecting the medical declaration of death and complicating end-of-life communication. This case illustrates how such reactions can be interpreted through cognitive dissonance theory. Recognizing these mechanisms provides a novel clinical insight into the need for structured, empathetic, and repeated communication strategies that explicitly address cognitive dissonance, particularly for family members in emotionally dominant roles, to reduce conflict, improve acceptance of brain death, and support ethically complex end-of-life discussions, including those related to organ donation.

## Introduction

Brain death, also referred to as death by neurologic criteria (BD/DNC), has been recognized for more than four decades as a medically and legally accepted definition of death [1]. It is defined as the irreversible cessation of all functions of the entire brain, including the brainstem, resulting in the complete and permanent loss of consciousness, spontaneous respiration, and brainstem reflexes [1,2,3]. The concept of "irreversibility" implies that neurologic function will not return spontaneously and that no medical intervention can restore brain activity [3]. From a legal standpoint, BD/DNC is considered equivalent to death determined by cardiopulmonary criteria [4].

The clinical determination of BD/DNC relies on a rigorous and systematic neurologic examination. The essential criteria include: (a) the presence of coma of known etiology, (b) the complete absence of all brainstem reflexes, and (c) failure of the apnea test [2,5]. It is equally important to exclude confounding factors such as hypothermia, metabolic disturbances, or drug intoxication that can mimic brain death [2,5]. Ancillary tests, such as electroencephalography (EEG), cerebral angiography, or radionuclide cerebral blood flow studies, may be used in selected circumstances but do not substitute for the clinical examination, which remains the cornerstone of diagnosis [2].

Despite its medical and legal standardization, BD/DNC often remains difficult for families to comprehend and accept. Modern medical technology, particularly mechanical ventilation, can maintain bodily functions such as heartbeat and temperature even after the definitive cessation of brain function. This apparent contradiction may generate profound cognitive dissonance, as the medical declaration of death conflicts with personal and cultural perceptions of life, producing significant emotional distress [2,4]. This perception highlights a fundamental contradiction, as the traditional image of death does not align with the image of brain death; the body continues to exhibit respiratory movements in the chest due to mechanical support, remains warm, and the heart continues to beat. Brain death, therefore, places relatives in a "gray zone" between life and death, making it extremely difficult for them to accept the condition of their loved one [6].

## Case presentation

A 65-year-old male was admitted to the intensive care unit (ICU) following a massive intracerebral hemorrhage. Despite maximal therapeutic interventions, his neurological status deteriorated rapidly, and clinical findings became strongly indicative of brain death. Neurological examination confirmed coma of known etiology with complete absence of brainstem reflexes, including pupillary light, corneal, oculocephalic, oculovestibular, gag, and cough reflexes. No supraspinal motor responses to painful stimuli were observed, with only spinally mediated reflexes present. Apnea testing, performed under standard prerequisites (temperature ≥36 °C, systolic blood pressure ≥100 mmHg, absence of hypoxemia and metabolic disturbances), demonstrated no spontaneous respiratory effort with arterial PaCO₂ exceeding 60 mmHg. These findings, together with the exclusion of confounding factors, confirmed the diagnosis of death by neurologic criteria in accordance with international guidelines (Table 1) [1].

**Table 1:** Clinical examination of the case study for the determination of brain death inspired by Greer et al. (2020) (Brain Death/ Death by Neurologic Criteria (BD/DNC)) Summary of the clinical examination components required for the neurologic determination of brain death, consistent with established guidelines [1]. BD/DNC = Brain Death/Death by Neurologic Criteria.

Examination step	Clinical findings of the case based on expected findings in BD/DNC	Important considerations
1. Pupillary assessment	The patient's pupils were mid-sized, remained fixed, and showed no reaction to light.	There were no confounding factors affecting the patient's pupil reactions, e.g., no ocular trauma or prior eye surgery, nor the presence of pharmacologic agents.
2. Corneal reflex	Corneal reflexes were absent bilaterally when the corneas were gently touched with a cotton swab; there was no eyelid movement, blink, or facial grimacing.	Gentle stimulation of the cornea was performed without applying excessive pressure on the globe. The complete absence of the corneal reflex was confirmed bilaterally.
3. Oculocephali c response	During passive head rotation (“doll’s eyes” maneuver), there was no deviation or independent movement of the eyes in any direction; the eyes remained fixed and moved passively with the head, without any counter-deviation, indicating the absence of the oculocephalic response.	There was no suspicion of cervical spine injury in this patient; therefore, the oculocephalic reflex test was safely performed, and the absence of any eye movement was reliably confirmed.
4. Oculovestibul ar response	With cold-water irrigation of each external auditory canal, there was no eye movement or tonic deviation of the eyes toward the irrigated ear, indicating the absence of oculovestibular responses. Tympanic membranes were confirmed intact, and ice-water irrigation was performed bilaterally.	Prior to testing, the integrity of both tympanic membranes was confirmed. Each external auditory canal was irrigated separately with ice water, and the complete absence of eye deviation was verified bilaterally.
5. Motor response to pain	Deep noxious stimulation failed to elicit any purposeful or non-purposeful motor activity; no spinal reflexes were observed in the limbs or face.	Deep noxious stimulation did not elicit any motor response. No spinal reflex movements (e.g., triple flexion) were observed, confirming the absence of spinal-mediated activity that could otherwise confound the clinical interpretation.
6. Gag and cough reflexes	Stimulation of the posterior pharynx produced no gag response, and tracheobronchial suctioning elicited no cough, indicating the absence of both gag and cough reflexes.	The appropriate catheter was correctly positioned during tracheobronchial suctioning, ensuring adequate stimulation. No factors were present that could interfere with the reflex arc (e.g., cervical spinal cord injury), making the absence of gag and cough reflexes reliable.
7. Apnea test	The apnea test was performed after completion of the full neurologic examination and under all required prerequisites (core temperature ≥36°C, systolic blood pressure ≥100 mmHg, and absence of hypoxemia or significant metabolic disturbances). During a controlled rise in PaCO₂, no spontaneous respiratory effort was observed. Arterial PaCO₂ increased to 79.4 mmHg with a corresponding pH of 7.18, confirming a positive apnea test consistent with BD/DNC.	The apnea test was safely completed without hypotension (blood pressure 120/67 mmHg), without hypoxemia (oxygen saturation 100%), and without arrhythmias (heart rate 85 bpm). The patient remained hemodynamically stable throughout the procedure, allowing for a complete and reliable apnea test without the need for ancillary testing.
Overall conclusion: Clinical examination and apnea test findings were consistent with BD/DNC, confirming the diagnosis.		

In line with institutional procedure, the confirmatory examinations were performed twice to validate the diagnosis of brain death. After completing these diagnostic steps, the patient's family, his wife and two children, a daughter and a son, were informed by the treating physician, together with the nurse coordinator for organ donation, in a private setting that ensured quiet and confidentiality.

The initial family meeting took place in the afternoon of the same day. A follow-up meeting was conducted the following morning to allow time for further discussion and questions. Initially, all family members asked questions about the patient's condition and the process of determining brain death. In contrast, the son reacted with disbelief and denial. This response may have been influenced by his perceived role as the primary protector of the patient, particularly as he appeared to assume increased responsibility following the anticipated loss of his father, as well as by strong pre-existing beliefs equating cardiac activity with life. In addition, observable expressions of personal or religious identity may have contributed to his interpretive framework, although these factors were not formally assessed. He focused on the fact that his father maintained a heartbeat, body warmth, and apparent "vitality" due to mechanical support, and he categorically refused to accept the declaration of death. This emotional conflict escalated into aggressive behavior, directed both at the team of physicians and nurses and at other family members; he engaged in verbal attacks and physical expressions of anger, such as hitting doors and objects in the ICU environment. As a result of the son's persistent denial and aggressive behavior, no space was left for meaningful dialogue regarding organ donation at that time.

Given the son's intense emotional reaction and the tension within the family, the team of physicians and nurses suggested a follow-up meeting the next day, allowing the relatives time to process the information and revisit questions. During this second meeting, the wife and daughter gradually showed greater understanding and acceptance of the diagnosis, in contrast to the son, who remained in denial and continued to reject the medical declaration of death. His persistent refusal and aggression further complicated the family's communication and delayed discussions regarding organ donation and end-of-life decisions.

This case highlights the profound psychological and emotional challenges that families may face when confronted with a brain death diagnosis. The apparent contradiction between medical findings and the loved one's external appearance can trigger a powerful mechanism of denial, which can be interpreted through the lens of cognitive dissonance theory.

## Discussion

In the present case, the son’s refusal to accept the diagnosis of brain death highlights the profound psychological and emotional challenges that families may experience when confronted with the apparent contradiction between medical findings and visible signs of bodily “life.” While the standardized neurological examination confirmed brain death beyond any doubt, the presence of cardiac activity, body warmth, and mechanically generated respiratory movements created the image of life, hence producing a strong sense of cognitive dissonance in the son.

The theory of cognitive dissonance, first introduced by Festinger (1957) [7], describes the psychological discomfort that arises when an individual simultaneously holds contradictory cognitions or beliefs [8,9]. The more central these beliefs are to one’s identity and values, the greater the intensity of dissonance [8,10]. In this case, the son’s conviction that his father was still “alive” because of apparent biological signs of vitality came into direct conflict with the medical declaration of irreversible death.

**Figure 1:**
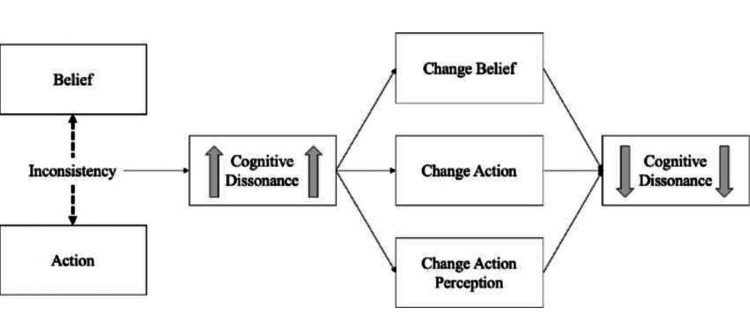
Cognitive dissonance resolution process Conceptual illustration based on Festinger’s theory of cognitive dissonance, depicting the psychological conflict arising from discrepant cognitions (e.g., observable bodily vitality versus medical determination of death) and potential pathways of dissonance reduction [7].

The progression of his behavior can be understood within the framework of dissonance-reduction strategies. Initially, the denial of the diagnosis functioned as a classic defense mechanism against an unbearable reality. Escalation to anger and aggression directed at the medical team and other family members can be interpreted as projection, whereby internal conflict is displaced onto external “culprits” [9,11]. Finally, his persistent belief that his father remained alive despite repeated explanations illustrates selective exposure to information, whereby discrepant medical explanations were rejected while visible physiological signs were privileged, reflecting the difficulty in resolving dissonance and leading to prolonged psychological distress [12,13] (Figure 1) [7].

The broader literature supports this interpretation, as families often struggle to understand the concept of brain death, frequently confusing it with coma or vegetative state, or perceiving signs such as cardiac activity as evidence of life [12,13,14]. Inadequate communication, compounded by the stress and exhaustion of the ICU environment, further impairs comprehension [13]. As Kompanje (2015) [12] notes, relatives require detailed, empathetic explanations, since conventional medical definitions of death do not align with what they observe at the bedside. Similarly, Sarti et al. (2023) [13] report that many families are unable to retain information provided during initial disclosure due to emotional overload.

The literature also highlights the importance of involving families in the diagnostic process. Allowing relatives to witness neurological testing may reduce denial and provide a sense of closure, whereas exclusion from the process is associated with distrust, prolonged grief, and negative psychological outcomes [13,14]. Moreover, families with a clearer understanding of brain death are more likely to provide mature and informed consent for organ donation [12,14].

Beyond the individual dimension, external and social factors also shape how families respond to brain death. Supportive communication frameworks, social validation, and group reassurance have been shown to reduce denial and facilitate trust [11,15,16]. Building on these insights, several practical strategies emerge from both this case and the literature. Clear and repeated communication, delivered in plain language, helps relatives retain information despite emotional overload [13]. Empathy and validation normalize distress as part of the grieving process. Involving families in the diagnostic process, such as by allowing them to witness neurological testing, may foster acceptance and closure [14]. Structured follow-up meetings provide time for processing before sensitive discussions, and interdisciplinary support from psychologists, social workers, or chaplains offers additional coping resources [12]. Together, these approaches can mitigate conflict, foster trust, and support healthier grieving. 

Finally, this case has the expected limitations of a single observation that cannot be generalized. The son’s personal background, health beliefs, and prior experiences were not systematically assessed, and cultural or religious factors were not formally examined. In such cases, personal religious beliefs can have an impact and may be a contributing factor in exacerbating cognitive dissonance. In addition, the specific wording and communication strategies used by the clinical team were not formally analyzed. In the future, such analyses would be worthwhile to consider.

Nevertheless, it illustrates that brain death is not only a medical diagnosis but also a profound symbolic event that generates intense cognitive and emotional conflict. Recognizing this dual nature underscores the ethical and professional responsibility of healthcare teams to support families, build trust, and facilitate acceptance of difficult end-of-life decisions.

## Conclusions

This case highlights the profound psychological and emotional challenges that families may face when confronted with a diagnosis of brain death. The son’s reaction illustrates how visible signs of bodily vitality can trigger cognitive dissonance, leading to denial, anger, and conflict with healthcare professionals. Understanding this response through the lens of cognitive dissonance theory underscores the need for healthcare teams to adopt empathetic, clear, and repeated communication strategies. The unique contribution of this case lies in framing aggressive or confrontational family behavior not as dissatisfaction with care, but as an expression of unresolved cognitive dissonance. This perspective has direct implications for clinical practice and communication training, emphasizing the importance of providing families with sufficient time, psychological safety, and structured opportunities to express and process conflicting beliefs. Involving families in the diagnostic process and providing ongoing emotional support can reduce distress, facilitate acceptance, and improve decision-making at the end of life. Ultimately, recognizing the psychological dimension of brain death determination is essential for fostering trust, guiding families through grief, and supporting ethically sensitive practices such as organ donation.
